# Physiological Functions of the β-Site Amyloid Precursor Protein Cleaving Enzyme 1 and 2

**DOI:** 10.3389/fnmol.2017.00097

**Published:** 2017-04-19

**Authors:** Riqiang Yan

**Affiliations:** Department of Neurosciences, Lerner Research Institute, Cleveland Clinic Foundation, ClevelandOH, USA

**Keywords:** Alzheimer’s disease, amyloid plaques, amyloid precursor protein, secretase, BACE1, BACE2, aspartic protease, BACE substrates

## Abstract

BACE1 was discovered as the β-secretase for initiating the cleavage of amyloid precursor protein (APP) at the β-secretase site, while its close homology BACE2 cleaves APP within the β-amyloid (Aβ) domain region and shows distinct cleavage preferences *in vivo*. Inhibition of BACE1 proteolytic activity has been confirmed to decrease Aβ generation and amyloid deposition, and thus specific inhibition of BACE1 by small molecules is a current focus for Alzheimer’s disease therapy. While BACE1 inhibitors are being tested in advanced clinical trials, knowledge regarding the properties and physiological functions of BACE is highly important and this review summarizes advancements in BACE1 research over the past several years. We and others have shown that BACE1 is not only a critical enzyme for testing the “Amyloid Hypothesis” associated with Alzheimer’s pathogenesis, but also important for various functions such as axon growth and pathfinding, astrogenesis, neurogenesis, hyperexcitation, and synaptic plasticity. BACE2 appears to play different roles such as glucose homeostasis and pigmentation. This knowledge regarding BACE1 functions is critical for monitoring the safe use of BACE1 inhibitors in humans.

## Introduction

For over 30 years, the study of β-amyloid (Aβ) peptides has been the largest field of research geared toward understanding Alzheimer’s pathogenesis and therapeutic intervention. After the molecular cloning of amyloid precursor protein (APP; Kang et al., 1987; Tanzi et al., 1987), it became clear that Aβ is a small fragment of APP that is located in the region partially spanning the transmembrane (TM) domain. The excision of Aβ from APP requires the sequential cleavage of APP by both β- and γ-secretase. In 1999, four groups independently reported identification of membrane-anchored aspartic protease as the β-secretase (Hussain et al., 1999; Sinha et al., 1999; Vassar et al., 1999; Yan et al., 1999), while the γ-secretase consists of presenilin-1 or -2, which forms a complex with additional multi-TM proteins nicastrin, pen2, and Aph1 (De Strooper et al., 1998; Wolfe et al., 1999; Li et al., 2000; Yu et al., 2000; Francis et al., 2002). After initial discovery, the β-secretase was named as BACE1, meaning β-site APP converting enzyme (Vassar et al., 1999). For the past 17 years, extensive efforts have been focused on the development of compounds that specifically inhibit BACE1 activity for Alzheimer’s disease (AD) therapy, and several major hurdles of producing brain-penetrable small molecular inhibitors have been overcome. Several highly potent BACE1 inhibitors have been developed by pharmaceutical and biotech companies and have been advanced to phase II/III clinical trials (see reviews by Ghosh and Osswald, 2014; Oehlrich et al., 2014; Vassar, 2014; Yan, 2016). Concurrently, BACE1 has also been shown to cleave multiple membrane substrates and its physiological roles in neuronal functions continue to be revealed (Vassar et al., 2014; Yan and Vassar, 2014; Hu et al., 2015a; Barao et al., 2016). Because of the importance of BACE1 inhibitors for therapeutic benefits in AD, this review will focus on summarizing the growing body of knowledge regarding the biological functions of BACE1.

## Bace1 is a Typical Aspartic Protease

In the initial molecular cloning of β-secretase, the Pharmacia group was exploring whether an aspartic protease functions as such a secretase (Yan et al., 1999). Two other groups had also screened for β-secretase activity through their aspartic protease collections (Hussain et al., 1999; Lin et al., 2000). Independently, all five groups demonstrated that the β-secretase is a type I TM aspartic protease having a classical bilobal structure with two active aspartate motifs (D_93_TG and D_289_SG). Although not broadly cited, this enzyme was also named to be memapsin 2 based on the standard nomenclature for aspartic proteases (Lin et al., 2000). The crystal structure of BACE1 shows gross similarity to other aspartic proteases, but the catalytic pocket is more open and less hydrophobic than that of other human aspartic proteases (Hong et al., 2000).

**FIGURE 1:**
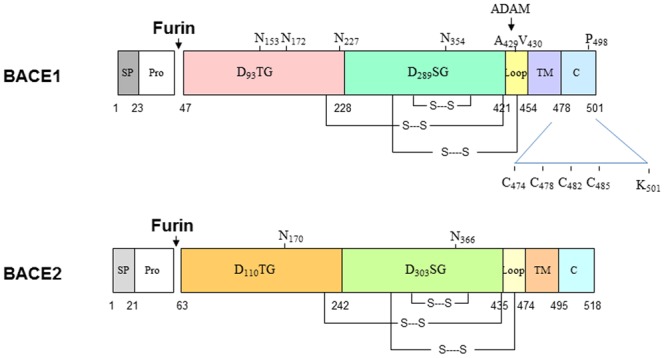
**Schematic illustration of BACE1 and BACE2 structural organizations.** Both BACE1 and BACE2 are type I transmembrane aspartic proteases, which have similar in length and 59% identify in the amino acid level. Both proteins are cleaved by furin to become mature form. BACE1 is also palmitoylated at C_474_, C_478_, C_482_, and C_485_; ubiquitinated at K_501_. Multiple lysine residues are suggested to be acetylated.

BACE1 is synthesized in the endoplasmic reticulum (ER) as a precursor protein having pre- (residues 1–21), pro- (residues 22–45), core protease- (residues 46–460), TM- (residues 461–477), and C-terminal domains (residues 478–501). BACE2 has 518 amino acids and almost identical structural organization (**Figure 1**). Both proteins share 59% identity and are two known aspartic proteases docked on the membrane through the type I TM domain (Hussain et al., 1999; Yan et al., 1999; Bennett et al., 2000; Lin et al., 2000). In the aspartic protease family, the prodomain usually assists in protein folding (Baker et al., 1993) and can flip to block the active pocket by conferring zymogen-like properties (Khan and James, 1998). Therefore, this prodomain is normally removed by furin-like proprotein convertases during maturation in the Golgi compartment to produce active enzyme. Interestingly, this prodomain has weak inhibitory effects and proBACE1 is enzymatically active (Shi et al., 2001). Consistent with this, BACE1 is active in the ER compartment (Yan et al., 2001a). While inhibiting prodomain removal has a weak effect on blocking BACE1 activity, enhancing shedding of BACE1 near the ectodomain region impacts its cleavage of APP. It is known that docking on the lipid bilayer is essential for BACE1 to cleave APP at the β-secretase site, as removing this TM domain abolishes cleavage of APP at the β-secretase site in cells (Yan et al., 2001a). This is consistent with observations that many soluble aspartic proteases cleave APP peptides at the β-secretase site *in vitro*, but not *in vivo* (Brown et al., 1996; Chevallier et al., 1997; Mackay et al., 1997; Gruninger-Leitch et al., 2000; Turner et al., 2002; Tomasselli et al., 2003).

BACE1 also undergoes other multiple post-translational modifications: it is N-glycosylated on four sites (N-153, N-172, N-223, and N254; Haniu et al., 2000), acetylated on seven Lys residues (Lys-126, Lys-275, Lys-279, Lys-285, Lys-299, Lys-300, and Lys-307) in the ER (Costantini et al., 2007), ubiquitinated at Lys-501 for the control of endocytosis to lysosomes for degradation (Tesco et al., 2007; Kang et al., 2012) and at Lys-203 and Lys-382 for the proteasomal degradation of BACE1 (Wang R. et al., 2012), palmitoylated in four C-terminal Cys residues (Cys474/478/482/485) for lipid raft localization (Benjannet et al., 2001; Vetrivel et al., 2009; Bhattacharyya et al., 2013), and phosphorylated at Ser-498 (Walter et al., 2001), which is linked to BACE1 cellular trafficking (Pastorino et al., 2002; He et al., 2005). Phosphorylation of BACE1 at Thr252 by the p25/Cdk5 complex appears to increase BACE1 activity (Song et al., 2015). A recent study suggests that glycol modifications of BACE1 by *N*-acetylglucosamine (GlcNAc), a sugar-bisecting enzyme highly expressed in brain, regulates BACE1 stability (Kizuka et al., 2015). Loss of GlcNAc will lead to enhanced degradation of BACE1 by increased trafficking of BACE1 to lysosomes from the late endosomes. This is reminiscent of deubiquitinylation by ubiquitin-specific peptidase 8 (USP8), an endosome-associated deubiquitinating enzyme. Studies have shown that RNAi-mediated depletion of USP8 increased BACE1 ubiquitination on Lys-501, promoted BACE1 accumulation in the early endosomes and late endosomes/lysosomes, and decreased levels of BACE1 in the recycling endosomes (Yeates and Tesco, 2016). It should be noted that most post-translational modifications, except for the disulfide linkage, can regulate BACE1 activity but are not necessary for BACE1 proteolytic activity *per se*, as recombinant BACE1 produced in bacteria lacks these modifications but is sufficiently active.

## Cellular Trafficking of Bace1

BACE1 is first synthesized in the ER and then is distributed to various cellular compartments such as the Golgi network, endosomes, and cell surface, where the luminal BACE1 catalytic domain will cleave its cellular substrates such as APP. Like other aspartic proteases, the catalytic activity of BACE1 is elevated in more acidic environments (Shimizu et al., 2008). Because of this preferential activation, altered localization or cellular trafficking of BACE1 in cellular compartments impacts generation of Aβ from the cleavage of APP (Vassar et al., 2009).

Several proteins have now been shown to bind BACE1 and to alter cellular localization. Golgi-localized γ-ears containing proteins from the ADP ribosylation factor-binding (GGA) family were first shown to bind to BACE1 via the dileucine motif, and this binding impacts not only BACE1 endosomal trafficking but also cellular stability (He et al., 2002, 2005; Wahle et al., 2005; Tesco et al., 2007; Santosa et al., 2011; Walker et al., 2012; von et al., 2015). Depletion of both GGA1 and GGA3 induces a rapid and robust elevation of BACE1, and such an effect is likely inhibited by flotillin, which can compete with GGA proteins for binding to the same dileucine motif in the BACE1 tail (John et al., 2014). Reticulon (RTN) proteins, mainly localized in the ER, have been shown to bind BACE1 and this binding induces retention of BACE1 in the ER, which has a relatively neutral pH environment and thus is less favorable for APP cleavage by BACE1 (Sharoar and Yan, 2017). On the other hand, increased trafficking of BACE1 to the more acidic endosomes by cellular trafficking proteins such as the Vps10p domain-sorting receptor sortilin (Finan et al., 2011), the small GTPase ADP ribosylation factor 6 (ARF6; Sannerud et al., 2011), Rab-GTPases Rab11 (Udayar et al., 2013), and Sorting nexin 12 (Zhao et al., 2012) results in significant increases in Aβ generation.

In neurons, BACE1 is also targeted to axons and presynaptic terminals (Kandalepas et al., 2013) and its axonal transport is regulated by altered levels of calsyntenin-1 (Steuble et al., 2012; Vagnoni et al., 2012), retromer vps35 (Wen et al., 2011; Wang C.L. et al., 2012), RTN3 (Deng et al., 2013), Rab11 and Eps15 homology domain proteins (Buggia-Prevot et al., 2013, 2014; Udayar et al., 2013). The enhanced localization of BACE1 at synaptic sites is suggested to increase release of Aβ by the synaptic terminals and directly facilitates amyloid deposition in AD patients (Sadleir et al., 2016).

## Identified Bace1 Substrates

BACE1 cleaves many cellular substrates other than APP, so its biological functions will be affected by altered cleavage of these substrates. Various biochemical and proteomic approaches have been employed to search for BACE1 substrates. Initially, optimal BACE1 cleavage sites were explored (Gruninger-Leitch et al., 2002; Turner et al., 2002; Tomasselli et al., 2003), but this effort together with the use of bioinformatic tools was not successful in determining potential substrates. Instead, several BACE1 substrates were identified through candidate-based characterizations. For example, neuregulin-1 (Nrg1) was identified via the finding that BACE1 plays a role in regulating myelination (Hu et al., 2006; Willem et al., 2006).

Using unbiased proteomic analysis of cultured media from cell lines with or without overexpression of BACE1, Selkoe and his colleagues reported 68 putative BACE1 substrates (Hemming et al., 2009). By using the developed secretome protein enrichment with click sugars (SPECS) method, Lichtenthaler and his colleagues identified 34 membrane-associated proteins as potential BACE1 substrates (Kuhn et al., 2012). This group has also compared cerebrospinal fluids from BACE1-null vs. wild-type mice using label-free quantitative proteomics, and they identified additional novel substrates while validating several previously reported substrates (Dislich et al., 2015). Among these reported BACE1 substrates, the proteins listed in **Table 1** have gained the most attention and/or are fully validated.

**Table 1:** Partial list of characterized BACE1 substrates.

Protein substrate	The substrate recognition site	Reference
APP	KM↓DAEFRHDSGY↓EVHHQK^∨^LVFFAEDVGSNK-*TM*	
CHL-l	WGDNDSIFQ↓DVIETRGRETAGLDDISTG-*TM*	Zhou et al., 2012
Delta-1	GYVCECARGYGGPNCQFLLPELPPGPAVVDL↓TEKLEGQGG	Hu et al., 2017
IL-1R2	PVTREDLHMDFKCVVHNTLSF↓QTLRTTVKE-*TM*	Kuhn et al., 2007
Jag1	KE↓ITDKIIDLVSKRDGNSSLIA↓AVAE^∨^VRVQRRPLKNR-*TM*	He et al., 2014
Jag2	LIQGAAHAIVAAITQRGNSSLLL↓AVTE^∨^VKVETVVTGGS-*TM*	He et al., 2014
Navβ2	IMNPPDRHRGHGKIHL↓QVL↓MEEPPERDST-*TM*	Gersbacher et al., 2010
Nrg1 (type I and III-β1α)	GDRCQNYVMASF^v^YKHLGIEF↓MEAEELYQKR-*TM*	Hu et al., 2008
Nrg1 (type III-β1α)	TTETNL↓QTAPKLSTSTSTTGT-*EGF-like domain*	Fleck et al., 2013
Nrg3	FLPKTDSILSDPTDHLGIEF↓MESEEVYQRQ-*TM*	Hu et al., 2013
PSGL-1	VTHKGIPMAASNL↓SVNYPVGAPDHISVKQC-*TM*	Lichtenthaler et al., 2003
Sez6	AASLDGFYNGRSL↓DVAKAPASSALDAAH-*TM*	Pigoni et al., 2016
Sez6L	ICKVNQDSFEHALEA↓EAAAESSLEGGNMA-*TM*	Pigoni et al., 2016
ST6Gal 1	SGMAVKEQSKPMQFEKAQ↓LTLAEYDSGKK-*TM*	Kitazume et al., 2003

*↓ BACE2 cleavage site. ^∨^ α-secretase cleavage site.*

## Biological Functions Attributable To Bace1-Cleavable Substrates

As outlined above, the list of BACE1 substrates has grown in recent years (**Table 1**). It is highly important to understand the biological functions associated with BACE1 cleavage of these individual substrates. The following sections summarize studies on this topic that have been published in recent years.

### Astrogenesis and Neurogenesis

BACE1 was found to cleave Jagged-1 (Jag1), a type I TM ligand for Notch receptors (Hu et al., 2013). BACE1 mainly cleaves Jag1 at the A_1050_–A_1051_ site near the TM domain (He et al., 2014), and abolished cleavage in BACE1-null mice causes elevation of full-length Jag1, which in turn enhances Notch activation by producing high levels of Notch intracellular domain (NICD; Hu et al., 2013). Notch is highly expressed during neonatal stages and then gradually declines, with persistent low levels of expression in adulthood. BACE1 and Jag1 expression concurrently have the exact same patterns: high levels in neonatal stages and gradual reduction thereafter. Such parallel expression patterns in early developmental stages imply indispensable roles during development. Indeed, BACE1 deficiency causes enhanced astrogenesis and reduced neurogenesis, which is restricted to the hippocampal dentate gyrus (Hu et al., 2013). BACE1 and Jag1 are mainly expressed by pyramidal neurons, while Notch is highly expressed in neural stem cells in the subgranular zone. In earlier studies, high NICD activity was shown to inhibit neurogenesis in the postnatal dentate gyrus and to act as a switch from neurogenesis to gliogenesis (Morrison et al., 2000; Breunig et al., 2007). Hence, it appears that BACE1 regulates Notch signaling, via cleavage of Jag1, to control proliferation and differentiation of multi-potent neural precursor cells into neurons or astrocytes in the early developmental hippocampus.

### Myelination and Remyelination

The role of BACE1 in the control of myelination during development and of remyelination in the adult appears to occur through its cleavage of Nrg1 (Fleck et al., 2012; Hu et al., 2015a). Nrg1, which is one of the largest genes in the human genome with 33 spliced isoforms due to specific uses of six different transcriptional initiation sites as well as multiple splicing isoforms, is typically recognized by the presence of exons coding for the epidermal growth factor (EGF)-like domain (Holmes et al., 1992; Chang et al., 1997). Although Nrg1 isoforms with six different membrane topology types are found in the brain, types I and III β1 Nrg1 isoforms are mainly expressed by neurons and have been established as BACE1 substrates (Hu et al., 2006, 2008; Willem et al., 2006; La et al., 2011; Fleck et al., 2013). BACE1 specifically cleaves Nrg1 at the F-M site, which is located 10 residues before the TM domain and is shared by both types I and III β1 isoforms (Hu et al., 2008; La et al., 2011; Fleck et al., 2013). These two Nrg1 isoforms can also be cleaved by ADAM10 and ADAM17 at the F-Y site, which is seven residues upstream of BACE1 cleavage site. After cleavage by either BACE1 or ADAM10/17, type I Nrg1 releases its N-terminal fragment (Nrg1-ntf) to the extracellular space, where Nrg1-ntf binds to ErbB receptors (ErbB2 and ErbB3 heterodimers and ErbB4 homodimers) on nearby cells in a paracrine fashion, while type III Nrg1-ntf, which remains tethered to the lipid bilayer due to a hydrophobic CRD in its N-terminus, signals to adjacent cells in a juxtacrine fashion (Warren et al., 2006).

Activated Nrg1 signaling, mainly initiated by type III Nrg1, is critical for optimal myelination: mice with reduced Nrg1 signaling activity exhibit hypomyelination of peripheral nerves during development (Michailov et al., 2004; Taveggia et al., 2005). BACE1-null mice also display hypomyelination in their sciatic nerves, which are typically ensheathed by Schwann cells (Hu et al., 2006; Willem et al., 2006). This phenocopy is not only seen in BACE1-null mice but also in zebrafish (van Bebber et al., 2013) and rat (Weber et al., 2017) knockout (KO) models, and is consistent with reduced Nrg1 activity, which leads to decreased downstream signaling events such as reduced Akt phosphorylation and transcriptional expression of myelin genes like myelin basic proteins. Although type III Nrg1 is abundantly expressed in brain neurons (Liu et al., 2011), it has less effect on entheathing axons in the central nervous system, and both Nrg1 heterozygous mice and BACE1-null mice display weak hypomyelination phenotype in central nerves (Hu et al., 2006; Taveggia et al., 2008). Hypomyelination was observed in BACE1-null optic nerves, but not in broad brain regions of BACE1-null mice (Hu et al., 2006).

Interestingly, inhibition of BACE1 produces more dramatic suppression of myelination in a co-culture myelination system than pan inhibition of ADAM proteases (Luo et al., 2011). This is likely related to the presence of an additional BACE1 cleavage site between L-Q, located 16 residues upstream of the EGF-like domain in type III Nrg1 (Fleck et al., 2013). Cleavage of type III Nrg1 at these two BACE1 sites will release the EGF-like domain for signaling through ErbB receptors. This observation further supports the importance of BACE1-dependent Nrg1 signaling in myelination.

When peripheral nerves are severely injured, myelin on proximal segments of damaged axons can be removed due to Wallerian degeneration and regrowing axons will be remyelinated by Schwann cells via contacting regenerating axons in the proximal band of Büngner. BACE1 has been shown to be indispensable for remyelination in nerve crush experiments, as remyelinated axons remained hypomyelinated in BACE1-null sciatic nerves (Hu et al., 2008). In nerve transplantation experiments, it has been further demonstrated that nerve injury induces expression of BACE1 in Schwann cells and that this increased expression of BACE1 in Schwann cells is required for remyelination (Hu et al., 2015b). nerve region leads to shortened internode as well as reduced nerve conduction. BACE1 is also required for initial and optimal remyelination of corpus callosum axons, as demonstrated in cuprizone-induced demyelination experiments (Treiber et al., 2012).

In peripheral nerves, BACE1 cleavages of type I Nrg1 in Schwann cells and type III Nrg1 in axons contribute to normal remyelination. This conclusion is supported by mouse genetic studies using conditional deletion of Nrg1 isoforms or transgenic mice overexpressing either type I or type III Nrg1 (Fricker et al., 2011, 2013; Stassart et al., 2013). These studies show that Schwann cell-derived type I Nrg1 is dispensable for developmental myelination and myelin maintenance, but is required for an autocrine signaling function for remyelination, as loss of Nrg1 expression in Schwann cells severely impairs remyelination after nerve crush. More recently, it is shown that BACE1 can cleave Jag1 and Delta1 in axons and Schwann cells, and abrogated cleavage of these two Notch ligands enhances Schwann cell proliferation (Hu et al., 2017). This abnormally increased Schwann cell density within the given sciatic Hence, by cleaving types I and III Nrg1 as well as Jag1/Delta1 in different cell types, BACE1 controls these two signaling pathways to regulate optimal myelination and remyelination.

### Epileptic Seizures

BACE1-null mice develop convulsive and spontaneous behavioral seizures in an age-dependent manner, beginning at a young age and becoming more frequent with aging (Kobayashi et al., 2008; Hitt et al., 2010; Hu et al., 2010). Long-sustained epileptic seizures in BACE1-null mice likely contribute to neuronal loss in the 2-year-old mouse hippocampus, although neurodegeneration in this region was not evident in young mice (Hu et al., 2010). The molecular mechanism underlying this epileptic seizure activity remains elusive, and cleavages of multiple BACE1 substrates may each contribute. It has been shown that BACE1 cleaves voltage-gated sodium channel β subunits (Kim et al., 2005; Wong et al., 2005; Huth et al., 2011). Voltage-gated sodium channels consist of a heterotrimeric complex of one 260 kDa α-subunit and one or two auxiliary β subunits (Catterall, 2000), and abolished cleavage in BACE1-null mice likely increases surface expression of ion-conducting, channel-forming α-subunits through cellular trafficking (Isom, 2002; Yu et al., 2005). Sodium channel Na_v_1.2 protein was found to be elevated in BACE1-null hippocampal mossy fiber regions (Hu et al., 2010; Kim et al., 2011). This increase is consistent with greater neuronal excitability, as manifested by more frequent firing with larger amplitude in BACE1-null brain slices and a significant shift of the inactivation curve in the direction of depolarization (Dominguez et al., 2005; Hu et al., 2010). However, the BACE1- and subsequently γ-secretase-cleaved β subunit is also known to enhance gene expression of Na_v_ α subunits (Kim et al., 2007) and BACE1 deficiency will reduce the level of Na_v_ α subunits (Kim et al., 2011). Pharmacological blockage of sodium channel activity was not sufficient to reduce seizure activities (Hitt et al., 2010). Hence, the altered activity of sodium channel activity is unlikely to be the only explanation for seizure activity.

BACE1 can also cleave KCNE1 and KCNE2, two auxiliary subunits of voltage-gated potassium channels (Sachse et al., 2013). Both KCNE1 and KCNE2 are expressed in brains and altered functions of these two proteins are linked to epileptic seizures (Goldman et al., 2009; Heron et al., 2010). On the other hand, BACE1 deficiency may cause epilepsy through a none-enzymatic mechanism, as BACE1 interacts with an M-current-producing KCNQ (Kv7) family member, resembling the function of a β-subunit (Hessler et al., 2015). The loss of M-current due to BACE1 deficiency enhances neuronal excitability, which could also contribute to epileptic seizures.

Another family of proteins, the seizure-related gene 6 (Sez6) and its family member Sez6L, was identified as BACE1 substrate through an unbiased proteomic approach and was recently validated as a strong substrate of BACE1 (Kuhn et al., 2012). Their levels in BACE1-null CSF are significantly lowered, reflecting the abrogated cleavage by BACE1 (Pigoni et al., 2016). Although Sez6 KO mice have not been shown to have seizures, Sez6 is suggested to be a susceptibility gene for febrile seizures (Mulley et al., 2011). It remains to understand whether the abolished cleavage of Sez6 in BACE1-null mice contributes to epileptic seizures. Taking all of these findings into consideration, it is reasonable to postulate that multiple factors, including epigenetic factors, contribute to epileptic seizures in BACE1-null mice, which display variable spiking patterns on electroencephalography (Hitt et al., 2010; Hu et al., 2010).

### Muscle Spindle Defects

Muscle spindles, which are composed of specialized intrafusal muscle fibers, are innervated by afferent axons extending from sensory neurons (Hunt, 1990). Nrg1 in sensory neurons transduces its signals through ErbB2/ErbB3 receptors in muscles to control the formation of muscle spindles (Andrechek et al., 2002; Hippenmeyer et al., 2002; Leu et al., 2003). BACE1 deficiency impairs coordinated muscle function between forelimbs and hindlimbs, resulting in a swaying walking pattern as well as a reduction in the number of muscle spindles (Cheret et al., 2013). Such an ambulatory defect, likely due to dysfunctional proprioception governed by muscle spindles, is more dramatic in newborns while less severe in BACE1-null adult mice or heterozygous mice (Cheret et al., 2013). This role of BACE1 in reduced muscle spindle maintenance is due to abrogated or reduced cleavage of Ig domain-containing type I β1 Nrg1 (IgNrg1β1) isoforms, which are preferentially expressed by proprioceptive sensory neurons and are sufficient to induce muscle spindle differentiation in animals (Hippenmeyer et al., 2002). Consistently, transgenic mice overexpressing IgNrg1β1 develop supernumerary muscle spindles (Rumsey et al., 2008). If wild-type mice are treated with the BACE1 inhibitor Ly2811376 for 29 days, up to 40% of muscle spindles are lost (Cheret et al., 2013). As discussed previously (Hu et al., 2015a), abolished cleavage of Nrg1 reduces the expression of transcription factors in the early growth response (Egr) family. Egr3, in particular, controls expression of the muscle spindle-specific genes necessary for forming muscle spindle fibers. Hence, BACE1-dependent type I IgNrg1β1 signaling is critical for motor coordination.

### Axonal Growth and Neuronal Migration Defects

The neural cell adhesion molecule close homolog of L1 (CHL1), which is a type I membrane protein and a component of Sema3A receptors, is a natural BACE1 substrate with identified cleavage sites located between Y_1086_ and E_1087_, 18 residues upstream of the TM domain (Kuhn et al., 2012; Zhou et al., 2012). After BACE1 cleavage, which is inducible by Sema3A, CHL1-ntf, and CHL1-ctf are released. The CHL1-ctf appears to induce growth cone collapse in thalamic neurons (Barao et al., 2015), while the soluble CHL1-ntf may interact with neuropilin-1 to influence axon guidance (Hitt et al., 2012; Kuhn et al., 2012; Zhou et al., 2012). BACE1-null mice show axon pathfinding defects with mistargeting olfactory sensory neuron projections to glomeruli in the olfactory bulb and a shortened and disorganized infrapyramidal bundle of the mossy fiber projection from the dentate gyrus to CA3 in the hippocampus (Rajapaksha et al., 2011; Cao et al., 2012; Hitt et al., 2012). It should also be noted that BACE1 and CHL1 are co-localized in the terminals of hippocampal mossy fibers, olfactory sensory neuron axons, and growth cones of primary hippocampal neurons, and that axonal defective phenotypes in BACE1-null mice and CHL1-null mice are correlated, confirming the importance of BACE1 cleavage of CHL1 in neuronal development.

### Synaptic Dysfunctions

The effects of BACE1 inhibition on APP- or Aβ-mediated synaptic functions has recently been summarized in a separate review (Yan et al., 2016). As mentioned above, BACE1 can cleave type I Nrg1, which is highly important for synaptic functions through the signaling of ErbB4 receptors in the brain (see recent comprehensive review by Mei and Nave, 2014). More relevantly, Nrg1 has been identified as a susceptible gene in schizophrenia, which is a disease of synaptic dysfunction (Stefansson et al., 2002) and BACE1-null mice display schizophrenia-like behaviors, which include positive (hyperactivity and pre-pulse inhibition), negative (social withdrawal), and panel (cognitive functions) behaviors (Savonenko et al., 2008). In recent years, altered functioning of Nrg3, a member of the Nrg gene family, has also been found in association with schizophrenia pathogenesis. Nrg3 is an identified BACE1 substrate (Hu et al., 2008), further showing the importance of BACE1-dependent Nrg1 signaling functions. While hypo-function of Nrg1 is linked to schizophrenia-like behaviors in BACE1-null mice, enhanced expression of either BACE1-cleaved Nrg1-ntf fragment or of full-length Nrg1 surprisingly also induces schizophrenia-like behaviors (Kato et al., 2010; Luo et al., 2013; Yin et al., 2013; Agarwal et al., 2014). This is in line with clinical observations that increased Nrg1 or ErbB4 transcripts and proteins are found in schizophrenia patients (Harrison and Law, 2006; Geddes et al., 2011), supporting the importance of balanced BACE1-cleaved Nrg1 in synaptic functions.

BACE1-null mice also exhibit other synaptic dysfunctions. By electrophysiological recording of brain slices, it was demonstrated that hippocampal activity-dependent long-term potentiation at mossy fibers to CA3 is impaired, while long-term depression is increased (Wang et al., 2008, 2014). Intriguingly, a recent study reported that mice treated with the BACE1 inhibitors SCH1682496 and LY2811376 show impaired cognitive functions (Filser et al., 2015). As the list of identified BACE1 substrates has continued to grow (Hemming et al., 2009; Kuhn et al., 2012; Dislich et al., 2015), many of these potential substrates have been shown to control synaptic plasticity and their roles in BACE1-dependent synaptic functions have begun to gain attention. One such example discussed earlier is Sez6, which has been shown to play a role in synaptic function (Gunnersen et al., 2007). In Sez6 KO mice, dendritic spines are significantly shorted and excitatory synapses are thinner in the deep-layer pyramidal neurons of the somatosensory cortex, showing the importance of Sez6 in forming dendritic arbors and controlling synaptic plasticity. More roles of other BACE1 substrates are likely to emerge over the coming years.

### Retinopathy

The role of BACE1 in retinal pathophysiology has gained increasing attention in recent years, as several BACE1 inhibitors have been found to cause retinal thinning, lipofuscin accumulation, and vascular dysfunction, which terminated clinical trials (Fielden et al., 2015). BACE1 was initially suggested to mediate these retinopathies in a report that BACE1-null mice were found to develop retinal thinning, apoptosis, reduced retinal vascular density, and an increase in age pigmentation and lipofuscin (Cai et al., 2012). The mechanism is linked to the BACE cleavage of vascular endothelial growth factor receptor (VEGFR1). However, the retinal phenotypes in BACE1-null mice are controversial and are not seen in all lines of BACE1-null mice or in BACE1-null rat (Fielden et al., 2015), suggesting possible off-target toxicity. While retinopathy is closely monitored in BACE1 inhibitor clinical trials, recent studies have shown that it is likely due to cross-inhibition of cathepsin D by BACE1 inhibitors (Zuhl et al., 2016). Likely, this side effect can be mitigated by developing BACE1 inhibitors with minimal off-target inhibition of other aspartic proteases such as cathepsin D and E, both of which are important in lysosomal functions.

On the other hand, specific inhibition of BACE1 is likely to benefit retinal functions, as BACE1 activity in retina is elevated in response to stress conditions such as mitochondrial respiratory inhibition or oxidative stress (Xiong et al., 2007). It has been suggested that changes in BACE1 expression appear earlier in the retina than in the brain and precede behavioral deficits, and abnormal expression of BACE1 in the retina appears to be an early pathological change in APP/PS-1 transgenic mice (Li et al., 2016). BACE in the adult retina is mostly present in the plexiform layers, consistent with localization of this enzyme to synaptic terminals (Xiong et al., 2007). In AD, Aβ levels are elevated in neurodegenerative retinas, and this potentially causes damage in retinal function in aging (Dentchev et al., 2003; Gupta et al., 2016; Masuzzo et al., 2016). In this sense, inhibition of BACE1 will be beneficial to retinal function.

## Bace2 Substrates and its Biological Functions

While BACE2 was discovered simultaneously with BACE1 (Vassar et al., 2014), the functional importance of BACE2 has emerged after the finding that BACE2 cleaves the pro-proliferative plasma membrane protein Tmem27 and PMEL (see summary in **Table 2**). In pancreatic MIN6 cells treated with a BACE2 inhibitor or siRNA, BACE2 was initially shown to mediate insulin receptor β-subunit (IRβ) expression and surface trafficking (Casas et al., 2010). A separate BACE2 silencing study in murine and human β cells reveals Tmem27, known to promote the preservation of functional β-cell mass, as a BACE2 substrate (Esterhazy et al., 2011). Mice with BACE2 deficiency have been shown to correlatively increase β-cell mass, and improved control of glucose homeostasis is associated with increased insulin levels. Hence, BACE2 inhibition should be beneficial to diabetic patients by controlling β-cell maintenance and glucose metabolism.

**Table 2:** Characterized BACE2 substrates.

Protein substrate	The substrate recognition site	Reference
APP	KMDAEFRHDSGYEVHHQK^∨^LVF↓F↓AEDVGSNK-*TM*	Farzan et al., 2000; Yan et al., 2001b
IAPP (human)	KCNTATCATQRLANF↓LVHSSNNF↓GAISSTNVGSNTY-*TM*	Rulifson et al., 2016
IAPP (rodent)	KCNTATCATQRLANF↓LVRSSNNLGPVLPPTNVGSNTY-*TM*	Rulifson et al., 2016
Jag1	KEITDKIIDL↓VSKRDGNSSLIA↓AVAE^∨^VRVQRRPLKNR-*TM*	He et al., 2014
Tmem27	RMNKNRINNAF↓FL↓NDQTLEF↓LKIPSTLAPP-*TM*	Esterhazy et al., 2012

*↓ BACE2 cleavage site. ^∨^ α-secretase cleavage site.*

To maintain glucose homeostasis, islet amyloid polypeptide (IAPP) in pancreatic β cells needs to co-secrete with insulin, and the formation of IAPP amyloid is a hallmark pathological feature of type 2 diabetes (Mukherjee et al., 2015). BACE2 was recently shown to cleave IAPP at two ectodomain sites (Rulifson et al., 2016) and loss of BACE2 cleavage likely increases IAPP homodimer formation and subsequent production of cytotoxic oligomers and amyloid fibrils. Hence, this study suggests that BACE2 inhibition may lead to β-cell dysfunction due to IAPP accumulation in proteinaceous plaques in and around pancreatic islets. These two controversial aspects will be further resolved in more detailed future studies.

On the other hand, BACE2 inhibition can cause loss of pigmentation, as BACE2 cleaves the integral membrane form of PMEL within the juxtamembrane domain and exerts its role in melanosome biogenesis (Rochin et al., 2013). Although BACE1 is expressed in pigment cells, the level of BACE2 is 37-fold higher as that in retinal pigment epithelial cells, suggesting that BACE2 is the major BACE homolog in pigment cells. Consistently, mice with BACE2 deficiency show loss of pigment in skin and retina. However, BACE2 depletion reduces neither the number of stage IV melanized melanosomes nor the total melanin content. Instead, the loss of BACE2-cleaved PMEL N-terminal fragment impairs the organization of PMEL fibrils into parallel sheets, with a threefold decrease in the number of fibrillar stage II and III melanosomes and a sixfold increase in the number of round organelles containing unstructured aggregates. Hence, BACE2 is required for the formation of PMEL amyloid fibrils and for melanosome morphogenesis, consistent with demonstrations by pharmacological inhibition of BACE1 and BACE2 (Shimshek et al., 2016).

It is also demonstrated that Sez6L and Sez6L2 are effectively cleaved but in rate limiting proteolytic manner in pancreatic islet β-cells by BACE2 (Stutzer et al., 2013). Although Sez6L is also a BACE1 substrate, it is not cleaved by BACE1 in pancreatic cells (Pigoni et al., 2016). Additional BACE2 substrates, explored through proteomic approaches, include CD200, IGF2R, LAMP2, MPZL1, and SORT1 (Stutzer et al., 2013). The functional importance of BACE2 cleavages of these proteins remains to be established.

## Summary

Inhibition of BACE1 is one the most promising therapeutic targets for treating AD, and five drugs have currently entered into clinical trials (Vassar, 2014; Yan, 2016). While there is great promise for BACE1 inhibition in benefiting Alzheimer’s patients, it also raises caution regarding mechanism-based side effects associated with long-lasting inhibition of this enzyme. In addition to the fact that BACE1 is indispensable for proper astrogenesis, axonal growth and migration, myelination and remyelination, neuronal excitation, and synaptic plasticity, BACE1-null mice are also found to be susceptible to early lethality (Dominguez et al., 2005; Weber et al., 2017). Reduced body weight is seen in BACE1-null mice but not in rats. With more efforts, the available BACE1-null mice and rats will identify more shared phenotypes, and these phenotypes will have to be taken into consideration when BACE1 is inhibited for long terms. Moreover, BACE1 inhibition may also cause cross-inhibition with BACE2, as some compounds such as MK8931 appear to be more potent in blocking BACE2 activity (see reviews by Yan, 2016). Although BACE1 and BACE2 exhibit distinct cleavage specificity, substrates like APP, Jag1 and Sez6 family proteins are shared by these two enzymes. The number of studies using BACE2-null mice is increasing, and inhibition of BACE2 may alter glucose homeostasis and pigmentation. Future studies are expected to provide more knowledge regarding the biological functions of BACE1 and BACE2 in brains and other tissues.

## Author Contributions

The author confirms being the sole contributor of this work and approved it for publication.

## Conflict of Interest Statement

The author declares that the research was conducted in the absence of any commercial or financial relationships that could be construed as a potential conflict of interest.

## References

[B1] Agarwal A. Zhang M. Trembak-Duff I. Unterbarnscheidt T. Radyushkin K. Dibaj P. (2014). Dysregulated expression of neuregulin-1 by cortical pyramidal neurons disrupts synaptic plasticity. *Cell Rep.* 8 1130–1145. 10.1016/j.celrep.2014.07.026 25131210

[B2] Andrechek E. R. Hardy W. R. Girgis-Gabardo A. A. Perry R. L. Butler R. Graham F. L. (2002). ErbB2 is required for muscle spindle and myoblast cell survival. *Mol. Cell. Biol.* 22 4714–4722. 10.1128/MCB.22.13.4714-4722.2002 12052879 PMC133917

[B3] Baker D. Shiau A. K. Agard D. A. (1993). The role of pro regions in protein folding. *Curr. Opin. Cell Biol.* 5 966–970. 10.1016/0955-0674(93)90078-5 8129949

[B4] Barao S. Gartner A. Leyva-Diaz E. Demyanenko G. Munck S. Vanhoutvin T. (2015). Antagonistic effects of BACE1 and APH1B-gamma-secretase control axonal guidance by regulating growth cone collapse. *Cell Rep.* 12 1367–1376. 10.1016/j.celrep.2015.07.059 26299962 PMC4820248

[B5] Barao S. Moechars D. Lichtenthaler S. F. De Strooper B. (2016). BACE1 physiological functions may limit its use as therapeutic target for Alzheimer’s disease. *Trends Neurosci.* 39 158–169. 10.1016/j.tins.2016.01.003 26833257

[B6] Benjannet S. Elagoz A. Wickham L. Mamarbachi M. Munzer J. S. Basak A. (2001). Post-translational processing of beta-secretase (beta-amyloid-converting enzyme) and its ectodomain shedding. The pro- and transmembrane/cytosolic domains affect its cellular activity and amyloid-beta production. *J. Biol. Chem.* 276 10879–10887. 10.1074/jbc.M009899200 11152688

[B7] Bennett B. D. Babu-Khan S. Loeloff R. Louis J. C. Curran E. Citron M. (2000). Expression analysis of BACE2 in brain and peripheral tissues. *J. Biol. Chem.* 275 20647–20651. 10.1074/jbc.M002688200 10749877

[B8] Bhattacharyya R. Barren C. Kovacs D. M. (2013). Palmitoylation of amyloid precursor protein regulates amyloidogenic processing in lipid rafts. *J. Neurosci.* 33 11169–11183. 10.1523/JNEUROSCI.4704-12.2013 23825420 PMC3718372

[B9] Breunig J. J. Silbereis J. Vaccarino F. M. Sestan N. Rakic P. (2007). Notch regulates cell fate and dendrite morphology of newborn neurons in the postnatal dentate gyrus. *Proc. Natl. Acad. Sci. U.S.A.* 104 20558–20563. 10.1073/pnas.0710156104 18077357 PMC2154470

[B10] Brown A. M. Tummolo D. M. Spruyt M. A. Jacobsen J. S. Sonnenberg-Reines J. (1996). Evaluation of cathepsins D and G and EC 3.4.24.15 as candidate beta-secretase proteases using peptide and amyloid precursor protein substrates. *J. Neurochem.* 66 2436–2445. 10.1046/j.1471-4159.1996.66062436.x 8632167

[B11] Buggia-Prevot V. Fernandez C. G. Riordan S. Vetrivel K. S. Roseman J. Waters J. (2014). Axonal BACE1 dynamics and targeting in hippocampal neurons: a role for Rab11 GTPase. *Mol. Neurodegener.* 9:1 10.1186/1750-1326-9-1 PMC4031619 24386896

[B12] Buggia-Prevot V. Fernandez C. G. Udayar V. Vetrivel K. S. Elie A. Roseman J. (2013). A function for EHD family proteins in unidirectional retrograde dendritic transport of BACE1 and Alzheimer’s disease Abeta production. *Cell Rep.* 5 1552–1563. 10.1016/j.celrep.2013.12.006 24373286 PMC3932704

[B13] Cai J. Qi X. Kociok N. Skosyrski S. Emilio A. Ruan Q. (2012). beta-Secretase (BACE1) inhibition causes retinal pathology by vascular dysregulation and accumulation of age pigment. *EMBO Mol. Med.* 4 980–991. 10.1002/emmm.201101084 22903875 PMC3491829

[B14] Cao L. Rickenbacher G. T. Rodriguez S. Moulia T. W. Albers M. W. (2012). The precision of axon targeting of mouse olfactory sensory neurons requires the BACE1 protease. *Sci. Rep.* 2:231 10.1038/srep00231 PMC3262176 22355745

[B15] Casas S. Casini P. Piquer S. Altirriba J. Soty M. Cadavez L. (2010). BACE2 plays a role in the insulin receptor trafficking in pancreatic beta-cells. *Am. J. Physiol. Endocrinol. Metab.* 299 E1087–E1095. 10.1152/ajpendo.00420.2010 20943756

[B16] Catterall W. A. (2000). From ionic currents to molecular mechanisms: the structure and function of voltage-gated sodium channels. *Neuron* 26 13–25. 10.1016/S0896-6273(00)81133-2 10798388

[B17] Chang H. Riese D. J. Gilbert W. Stern D. F. McMahan U. J. (1997). Ligands for ErbB-family receptors encoded by a neuregulin-like gene. *Nature* 387 509–512. 10.1038/387509a0 9168114

[B18] Cheret C. Willem M. Fricker F. R. Wende H. Wulf-Goldenberg A. Tahirovic S. (2013). Bace1 and Neuregulin-1 cooperate to control formation and maintenance of muscle spindles. *EMBO J.* 32 2015–2028. 10.1038/emboj.2013.146 23792428 PMC3715864

[B19] Chevallier N. Vizzavona J. Marambaud P. Baur C. P. Spillantini M. Fulcrand P. (1997). Cathepsin D displays in vitro beta-secretase-like specificity. *Brain Res.* 750 11–19. 10.1016/S0006-8993(96)01330-3 9098524

[B20] Costantini C. Ko M. H. Jonas M. C. Puglielli L. (2007). A reversible form of lysine acetylation in the ER and Golgi lumen controls the molecular stabilization of BACE1. *Biochem. J.* 407 383–395. 10.1042/BJ20070040 17425515 PMC2275071

[B21] De Strooper B. Saftig P. Craessaerts K. Vanderstichele H. Guhde G. Annaert W. (1998). Deficiency of presenilin-1 inhibits the normal cleavage of amyloid precursor protein. *Nature* 391 387–390. 10.1038/34910 9450754

[B22] Deng M. He W. Tan Y. Han H. Hu X. Xia K. (2013). Increased expression of reticulon 3 in neurons leads to reduced axonal transport of beta site amyloid precursor protein-cleaving enzyme 1. *J. Biol. Chem.* 288 30236–30245. 10.1074/jbc.M113.480079 24005676 PMC3798490

[B23] Dentchev T. Milam A. H. Lee V. M. Trojanowski J. Q. Dunaief J. L. (2003). Amyloid-beta is found in drusen from some age-related macular degeneration retinas, but not in drusen from normal retinas. *Mol. Vis.* 9 184–190. 12764254

[B24] Dislich B. Wohlrab F. Bachhuber T. Mueller S. Kuhn P. H. Hogl S. (2015). Label-free quantitative proteomics of mouse cerebrospinal fluid detects BACE1 protease substrates in vivo. *Mol. Cell. Proteomics* 14 2550–2563. 10.1074/mcp.M114.041533 26139848 PMC4597136

[B25] Dominguez D. Tournoy J. Hartmann D. Huth T. Cryns K. Deforce S. (2005). Phenotypical and biochemical analysis of BACE1 and BACE2 deficient mice. *J. Biol. Chem.* 280 30797–30806. 10.1074/jbc.M505249200 15987683

[B26] Esterhazy D. Akpinar P. Stoffel M. (2012). Tmem27 dimerization, deglycosylation, plasma membrane depletion, and the extracellular Phe-Phe motif are negative regulators of cleavage by Bace2. *Biol. Chem.* 393 473–484. 10.1515/hsz-2012-0104 22628310

[B27] Esterhazy D. Stutzer I. Wang H. Rechsteiner M. P. Beauchamp J. Dobeli H. (2011). Bace2 is a beta cell-enriched protease that regulates pancreatic beta cell function and mass. *Cell Metab.* 14 365–377. 10.1016/j.cmet.2011.06.018 21907142

[B28] Farzan M. Schnitzler C. E. Vasilieva N. Leung D. Choe H. (2000). BACE2, a β-secretase homolog, cleaves at the β site and within the amyloid-β region of the amyloid-β precursor protein. *Proc. Natl. Acad. Sci. U.S.A.* 97 9712–9717. 10.1073/pnas.160115697 10931940 PMC16930

[B29] Fielden M. R. Werner J. Jamison J. A. Coppi A. Hickman D. Dunn R. T. (2015). Retinal toxicity induced by a novel beta-secretase inhibitor in the sprague-dawley rat. *Toxicol. Pathol.* 43 581–592. 10.1177/0192623314553804 25361751

[B30] Filser S. Ovsepian S. V. Masana M. Blazquez-Llorca L. Brandt E. A. Volbracht C. (2015). Pharmacological inhibition of BACE1 impairs synaptic plasticity and cognitive functions. *Biol. Psychiatry* 77 729–739. 10.1016/j.biopsych.2014.10.013 25599931

[B31] Finan G. M. Okada H. Kim T. W. (2011). BACE1 retrograde trafficking is uniquely regulated by the cytoplasmic domain of sortilin. *J. Biol. Chem.* 286 12602–12616. 10.1074/jbc.M110.170217 21245145 PMC3069461

[B32] Fleck D. Garratt A. N. Haass C. Willem M. (2012). BACE1 dependent neuregulin processing: review. *Curr. Alzheimer Res.* 9 178–183. 10.2174/156720512799361637 22455478

[B33] Fleck D. van Bebber F. Colombo A. Galante C. Schwenk B. M. Rabe L. (2013). Dual cleavage of neuregulin 1 type III by BACE1 and ADAM17 liberates its EGF-like domain and allows paracrine signaling. *J. Neurosci.* 33 7856–7869. 10.1523/JNEUROSCI.3372-12.2013 23637177 PMC6618983

[B34] Francis R. McGrath G. Zhang J. Ruddy D. A. Sym M. Apfeld J. (2002). aph-1 and pen-2 are required for Notch pathway signaling, gamma-secretase cleavage of betaAPP, and presenilin protein accumulation. *Dev. Cell* 3 85–97. 10.1016/S1534-5807(02)00189-2 12110170

[B35] Fricker F. R. Antunes-Martins A. Galino J. Paramsothy R. La R. F. Perkins J. (2013). Axonal neuregulin 1 is a rate limiting but not essential factor for nerve remyelination. *Brain* 136 2279–2297. 10.1093/brain/awt148 23801741 PMC3692042

[B36] Fricker F. R. Lago N. Balarajah S. Tsantoulas C. Tanna S. Zhu N. (2011). Axonally derived neuregulin-1 is required for remyelination and regeneration after nerve injury in adulthood. *J. Neurosci.* 31 3225–3233. 10.1523/JNEUROSCI.2568-10.2011 21368034 PMC3059576

[B37] Geddes A. E. Huang X.-F. Newell K. A. (2011). Reciprocal signalling between NR2 subunits of the NMDA receptor and neuregulin1 and their role in schizophrenia. *Prog. Neuropsychopharmacol. Biol. Psychiatry* 35 896–904. 10.1016/j.pnpbp.2011.02.017 21371516

[B38] Gersbacher M. T. Kim D. Y. Bhattacharyya R. Kovacs D. M. (2010). Identification of BACE1 cleavage sites in human voltage-gated sodium channel beta 2 subunit. *Mol. Neurodegener.* 5:61 10.1186/1750-1326-5-61 PMC3022600 21182789

[B39] Ghosh A. K. Osswald H. L. (2014). BACE1 (beta-secretase) inhibitors for the treatment of Alzheimer’s disease. *Chem. Soc. Rev.* 43 6765–6813. 10.1039/c3cs60460h 24691405 PMC4159447

[B40] Goldman A. M. Glasscock E. Yoo J. Chen T. T. Klassen T. L. Noebels J. L. (2009). Arrhythmia in heart and brain: KCNQ1 mutations link epilepsy and sudden unexplained death. *Sci. Transl. Med.* 1 2ra6 10.1126/scitranslmed.3000289 PMC2951754 20368164

[B41] Gruninger-Leitch F. Berndt P. Langen H. Nelboeck P. Dobeli H. (2000). Identification of beta-secretase-like activity using a mass spectrometry-based assay system. *Nat. Biotechnol.* 18 66–70. 10.1038/71944 10625394

[B42] Gruninger-Leitch F. Schlatter D. Kung E. Nelbock P. Dobeli H. (2002). Substrate and inhibitor profile of BACE (beta-secretase) and comparison with other mammalian aspartic proteases. *J. Biol. Chem.* 277 4687–4693. 10.1074/jbc.M109266200 11741910

[B43] Gunnersen J. M. Kim M. H. Fuller S. J. De Silva M. Britto J. M. Hammond V. E. (2007). Sez-6 proteins affect dendritic arborization patterns and excitability of cortical pyramidal neurons. *Neuron* 56 621–639. 10.1016/j.neuron.2007.09.018 18031681

[B44] Gupta V. Gupta V. B. Chitranshi N. Gangoda S. Vander W. R. Abbasi M. (2016). One protein, multiple pathologies: multifaceted involvement of amyloid beta in neurodegenerative disorders of the brain and retina. *Cell Mol. Life Sci.* 73 4279–4297. 10.1007/s00018-016-2295-x 27333888 PMC11108534

[B45] Haniu M. Denis P. Young Y. Mendiaz E. A. Fuller J. Hui J. O. (2000). Characterization of Alzheimer’s beta -secretase protein BACE. A pepsin family member with unusual properties. *J. Biol. Chem.* 275 21099–21106. 10.1074/jbc.M002095200 10887202

[B46] Harrison P. J. Law A. J. (2006). Neuregulin 1 and schizophrenia: genetics, gene expression, and neurobiology. *Biol. Psychiatry* 60 132–140. 10.1016/j.biopsych.2005.11.002 16442083

[B47] He W. Hu J. Xia Y. Yan R. (2014). beta-site amyloid precursor protein cleaving enzyme 1(BACE1) regulates Notch signaling by controlling the cleavage of Jagged 1 (Jag1) and Jagged 2 (Jag2) proteins. *J. Biol. Chem.* 289 20630–20637. 10.1074/jbc.M114.579862 24907271 PMC4110275

[B48] He X. Chang W. P. Koelsch G. Tang J. (2002). Memapsin 2 (beta-secretase) cytosolic domain binds to the VHS domains of GGA1 and GGA2: implications on the endocytosis mechanism of memapsin 2. *FEBS Lett.* 524 183–187. 10.1016/S0014-5793(02)03052-1 12135764

[B49] He X. Li F. Chang W. P. Tang J. (2005). GGA proteins mediate the recycling pathway of memapsin 2 (BACE). *J. Biol. Chem.* 280 11696–11703. 10.1074/jbc.M411296200 15615712

[B50] Hemming M. L. Elias J. E. Gygi S. P. Selkoe D. J. (2009). Identification of beta-secretase (BACE1) substrates using quantitative proteomics. *PLoS ONE* 4:e8477 10.1371/journal.pone.0008477 PMC2793532 20041192

[B51] Heron S. E. Hernandez M. Edwards C. Edkins E. Jansen F. E. Scheffer I. E. (2010). Neonatal seizures and long QT syndrome: a cardiocerebral channelopathy? *Epilepsia* 51 293–296. 10.1111/j.1528-1167.2009.02317.x 19863579

[B52] Hessler S. Zheng F. Hartmann S. Rittger A. Lehnert S. Volkel M. (2015). beta-Secretase BACE1 regulates hippocampal and reconstituted M-currents in a beta-subunit-like fashion. *J. Neurosci.* 35 3298–3311. 10.1523/JNEUROSCI.3127-14.2015 25716831 PMC6605557

[B53] Hippenmeyer S. Shneider N. A. Birchmeier C. Burden S. J. Jessell T. M. Arber S. (2002). A role for neuregulin1 signaling in muscle spindle differentiation. *Neuron* 36 1035–1049. 10.1016/S0896-6273(02)01101-7 12495620

[B54] Hitt B. D. Jaramillo T. C. Chetkovich D. M. Vassar R. (2010). BACE1-/- mice exhibit seizure activity that does not correlate with sodium channel level or axonal localization. *Mol. Neurodegener.* 5:31 10.1186/1750-1326-5-31 PMC2933677 20731874

[B55] Hitt B. Riordan S. M. Kukreja L. Eimer W. A. Rajapaksha T. W. Vassar R. (2012). β-site amyloid precursor protein (APP)-cleaving enzyme 1 (BACE1)-deficient mice exhibit a close homolog of L1 (CHL1) loss-of-function phenotype involving axon guidance defects. *J. Biol. Chem.* 287 38408–38425. 10.1074/jbc.M112.415505 22988240 PMC3493884

[B56] Holmes W. E. Sliwkowski M. X. Akita R. W. Henzel W. J. Lee J. Park J. W. (1992). Identification of heregulin, a specific activator of p185erbB2. *Science* 256 1205–1210. 10.1126/science.256.5060.1205 1350381

[B57] Hong L. Koelsch G. Lin X. Wu S. Terzyan S. Ghosh A. K. (2000). Structure of the protease domain of memapsin 2 (beta-secretase) complexed with inhibitor. *Science* 290 150–153. 10.1126/science.290.5489.150 11021803

[B58] Hu X. Fan Q. Hou H. Yan R. (2015a). Neurological dysfunctions associated with altered BACE1-dependent Neuregulin-1 signaling. *J. Neurochem.* 136 234–249. 10.1111/jnc.13395 26465092 PMC4833723

[B59] Hu X. He W. Diaconu C. Tang X. Kidd G. J. Macklin W. B. (2008). Genetic deletion of BACE1 in mice affects remyelination of sciatic nerves. *FASEB J.* 22 2970–2980. 10.1096/fj.08-106666 18413858 PMC2493455

[B60] Hu X. He W. Luo X. Tsubota K. E. Yan R. (2013). BACE1 regulates hippocampal astrogenesis via the Jagged1-Notch pathway. *Cell Rep.* 4 40–49. 10.1016/j.celrep.2013.06.005 23831026 PMC3740554

[B61] Hu X. Hicks C. W. He W. Wong P. Macklin W. B. Trapp B. D. (2006). Bace1 modulates myelination in the central and peripheral nervous system. *Nat. Neurosci.* 9 1520–1525. 10.1038/nn1797 17099708

[B62] Hu X. Hou H. Bastian C. He W. Qiu S. Ge Y. (2017). BACE1 regulates the proliferation and cellular functions of Schwann cells. *Glia* 65 712–726. 10.1002/glia.23122 28191691 PMC5357169

[B63] Hu X. Hu J. Dai L. Trapp B. Yan R. (2015b). Axonal and Schwann cell BACE1 is equally required for remyelination of peripheral nerves. *J. Neurosci.* 35 3806–3814. 10.1523/JNEUROSCI.5207-14.2015 25740511 PMC4348183

[B64] Hu X. Zhou X. He W. Yang J. Xiong W. Wong P. (2010). BACE1 deficiency causes altered neuronal activity and neurodegeneration. *J. Neurosci.* 30 8819–8829. 10.1523/JNEUROSCI.1334-10.2010 20592204 PMC2902368

[B65] Hunt C. C. (1990). Mammalian muscle spindle: peripheral mechanisms. *Physiol. Rev.* 70 643–663. 2194221 10.1152/physrev.1990.70.3.643

[B66] Hussain I. Powell D. Howlett D. R. Tew D. G. Meek T. D. Chapman C. (1999). Identification of a novel aspartic protease (Asp 2) as beta-secretase. *Mol. Cell. Neurosci.* 14 419–427. 10.1006/mcne.1999.0811 10656250

[B67] Huth T. Rittger A. Saftig P. Alzheimer C. (2011). beta-Site APP-cleaving enzyme 1 (BACE1) cleaves cerebellar Na+ channel beta4-subunit and promotes Purkinje cell firing by slowing the decay of resurgent Na+ current. *Pflugers. Arch.* 461 355–371. 10.1007/s00424-010-0913-2 21246381

[B68] Isom L. L. (2002). Beta subunits: players in neuronal hyperexcitability? *Novartis Found Symp.* 241 124–138. 11771642

[B69] John B. A. Meister M. Banning A. Tikkanen R. (2014). Flotillins bind to the dileucine sorting motif of beta-site amyloid precursor protein-cleaving enzyme 1 and influence its endosomal sorting. *FEBS J.* 281 2074–2087. 10.1111/febs.12763 24612608

[B70] Kandalepas P. C. Sadleir K. R. Eimer W. A. Zhao J. Nicholson D. A. Vassar R. (2013). The Alzheimer’s beta-secretase BACE1 localizes to normal presynaptic terminals and to dystrophic presynaptic terminals surrounding amyloid plaques. *Acta Neuropathol.* 126 329–352. 10.1007/s00401-013-1152-3 23820808 PMC3753469

[B71] Kang E. L. Biscaro B. Piazza F. Tesco G. (2012). BACE1 protein endocytosis and trafficking are differentially regulated by ubiquitination at lysine 501 and the Di-leucine motif in the carboxyl terminus. *J. Biol. Chem.* 287 42867–42880. 10.1074/jbc.M112.407072 23109336 PMC3522283

[B72] Kang J. Lemaire H. G. Unterbeck A. Salbaum J. M. Masters C. L. Grzeschik K. H. (1987). The precursor of Alzheimer’s disease amyloid A4 protein resembles a cell-surface receptor. *Nature* 325 733–736. 10.1038/325733a0 2881207

[B73] Kato T. Kasai A. Mizuno M. Fengyi L. Shintani N. Maeda S. (2010). Phenotypic characterization of transgenic mice overexpressing neuregulin-1. *PLoS ONE* 5:e14185 10.1371/journal.pone.0014185 PMC3000321 21151609

[B74] Khan A. R. James M. N. (1998). Molecular mechanisms for the conversion of zymogens to active proteolytic enzymes. *Protein Sci.* 7 815–836. 10.1002/pro.5560070401 9568890 PMC2143990

[B75] Kim D. Y. Carey B. W. Wang H. Ingano L. A. Binshtok A. M. Wertz M. H. (2007). BACE1 regulates voltage-gated sodium channels and neuronal activity. *Nat. Cell Biol.* 9 755–764. 10.1038/ncb1602 17576410 PMC2747787

[B76] Kim D. Y. Gersbacher M. T. Inquimbert P. Kovacs D. M. (2011). Reduced sodium channel Na(v)1.1 levels in BACE1-null mice. *J. Biol. Chem.* 286 8106–8116. 10.1074/jbc.M110.134692 21190943 PMC3048697

[B77] Kim D. Y. Ingano L. A. Carey B. W. Pettingell W. H. Kovacs D. M. (2005). Presenilin/gamma-secretase-mediated cleavage of the voltage-gated sodium channel beta2-subunit regulates cell adhesion and migration. *J. Biol. Chem.* 280 23251–23261. 10.1074/jbc.M412938200 15833746

[B78] Kitazume S. Tachida Y. Oka R. Kotani N. Ogawa K. Suzuki M. (2003). Characterization of α2,6-sialyltransferase cleavage by Alzheimer’s β-secretase (BACE1). *J. Biol. Chem.* 278 14865–14871. 10.1074/jbc.M206262200 12473667

[B79] Kizuka Y. Kitazume S. Fujinawa R. Saito T. Iwata N. Saido T. C. (2015). An aberrant sugar modification of BACE1 blocks its lysosomal targeting in Alzheimer’s disease. *EMBO Mol. Med.* 7 175–189. 10.15252/emmm.201404438 25592972 PMC4328647

[B80] Kobayashi D. Zeller M. Cole T. Buttini M. McConlogue L. Sinha S. (2008). BACE1 gene deletion: impact on behavioral function in a model of Alzheimer’s disease. *Neurobiol. Aging* 29 861–873. 10.1016/j.neurobiolaging.2007.01.002 17331621

[B81] Kuhn P. H. Koroniak K. Hogl S. Colombo A. Zeitschel U. Willem M. (2012). Secretome protein enrichment identifies physiological BACE1 protease substrates in neurons. *EMBO J.* 31 3157–3168. 10.1038/emboj.2012.173 22728825 PMC3400020

[B82] Kuhn P. H. Marjaux E. Imhof A. De Strooper B. Haass C. Lichtenthaler S. F. (2007). Regulated intramembrane proteolysis of the interleukin-1 receptor II by α-, β-, and γ-secretase. *J. Biol. Chem.* 282 11982–11995. 10.1074/jbc.M700356200 17307738

[B83] La M. R. Cerri F. Horiuchi K. Bachi A. Feltri M. L. Wrabetz L. (2011). TACE (ADAM17) inhibits Schwann cell myelination. *Nat. Neurosci.* 14 857–865. 10.1038/nn.2849 21666671 PMC3291894

[B84] Leu M. Bellmunt E. Schwander M. Farinas I. Brenner H. R. Muller U. (2003). Erbb2 regulates neuromuscular synapse formation and is essential for muscle spindle development. *Development* 130 2291–2301. 10.1242/dev.00447 12702645

[B85] Li L. Luo J. Chen D. Tong J. B. Zeng L. P. Cao Y. Q. (2016). BACE1 in the retina: a sensitive biomarker for monitoring early pathological changes in Alzheimer’s disease. *Neural Regen. Res.* 11 447–453. 10.4103/1673-5374.179057 27127484 PMC4829010

[B86] Li Y. M. Xu M. Lai M. T. Huang Q. Castro J. L. DiMuzio-Mower J. (2000). Photoactivated gamma-secretase inhibitors directed to the active site covalently label presenilin 1. *Nature* 405 689–694. 10.1038/35015085 10864326

[B87] Lichtenthaler S. F. Dominguez D. I. Westmeyer G. G. Reiss K. Haass C. Saftig P. (2003). The cell adhesion protein P-selectin glycoprotein ligand-1 is a substrate for the aspartyl protease BACE1. *J. Biol. Chem.* 278 48713–48719. 10.1074/jbc.M303861200 14507929

[B88] Lin X. Koelsch G. Wu S. Downs D. Dashti A. Tang J. (2000). Human aspartic protease memapsin 2 cleaves the beta-secretase site of beta-amyloid precursor protein. *Proc. Natl. Acad. Sci. U.S.A.* 97 1456–1460. 10.1073/pnas.97.4.1456 10677483 PMC26455

[B89] Liu X. Bates R. Yin D. M. Shen C. Wang F. Su N. (2011). Specific regulation of NRG1 isoform expression by neuronal activity. *J. Neurosci.* 31 8491–8501. 10.1523/JNEUROSCI.5317-10.2011 21653853 PMC3154699

[B90] Luo X. He W. Hu X. Yan R. (2013). Reversible overexpression of bace1-cleaved neuregulin-1 N-terminal fragment induces schizophrenia-like phenotypes in mice. *Biol. Psychiatry* 76 120–127. 10.1016/j.biopsych.2013.09.026 24210810 PMC3976896

[B91] Luo X. Prior M. He W. Hu X. Tang X. Shen W. (2011). Cleavage of neuregulin-1 by BACE1 or ADAM10 protein produces differential effects on myelination. *J. Biol. Chem.* 286 23967–23974. 10.1074/jbc.M111.251538 21576249 PMC3129178

[B92] Mackay E. A. Ehrhard A. Moniatte M. Guenet C. Tardif C. Tarnus C. (1997). A possible role for cathepsins D., E., and B in the processing of beta-amyloid precursor protein in Alzheimer’s disease. *Eur. J. Biochem.* 244 414–425. 10.1111/j.1432-1033.1997.00414.x 9119007

[B93] Masuzzo A. Dinet V. Cavanagh C. Mascarelli F. Krantic S. (2016). Amyloidosis in retinal neurodegenerative diseases. *Front. Neurol.* 7:127 10.3389/fneur.2016.00127 PMC4976396 27551275

[B94] Mei L. Nave K. A. (2014). Neuregulin-ERBB signaling in the nervous system and neuropsychiatric diseases. *Neuron* 83 27–49. 10.1016/j.neuron.2014.06.007 24991953 PMC4189115

[B95] Michailov G. V. Sereda M. W. Brinkmann B. G. Fischer T. M. Haug B. Birchmeier C. (2004). Axonal neuregulin-1 regulates myelin sheath thickness. *Science* 304 700–703. 10.1126/science.1095862 15044753

[B96] Morrison S. J. Perez S. E. Qiao Z. Verdi J. M. Hicks C. Weinmaster G. (2000). Transient Notch activation initiates an irreversible switch from neurogenesis to gliogenesis by neural crest stem cells. *Cell* 101 499–510. 10.1016/S0092-8674(00)80860-0 10850492

[B97] Mukherjee A. Morales-Scheihing D. Butler P. C. Soto C. (2015). Type 2 diabetes as a protein misfolding disease. *Trends Mol. Med.* 21 439–449. 10.1016/j.molmed.2015.04.005 25998900 PMC4492843

[B98] Mulley J. C. Iona X. Hodgson B. Heron S. E. Berkovic S. F. Scheffer I. E. (2011). The role of seizure-related SEZ6 as a susceptibility gene in febrile seizures. *Neurol. Res. Int.* 2011:917565 10.1155/2011/917565 PMC3139179 21785725

[B99] Oehlrich D. Prokopcova H. Gijsen H. J. (2014). The evolution of amidine-based brain penetrant BACE1 inhibitors. *Bioorg. Med. Chem. Lett.* 24 2033–2045. 10.1016/j.bmcl.2014.03.025 24704031

[B100] Pastorino L. Ikin A. F. Nairn A. C. Pursnani A. Buxbaum J. D. (2002). The carboxyl-terminus of BACE contains a sorting signal that regulates BACE trafficking but not the formation of total A(beta). *Mol. Cell. Neurosci.* 19 175–185. 10.1006/mcne.2001.1065 11860271

[B101] Pigoni M. Wanngren J. Kuhn P. H. Munro K. M. Gunnersen J. M. Takeshima H. (2016). Seizure protein 6 and its homolog seizure 6-like protein are physiological substrates of BACE1 in neurons. *Mol. Neurodegener.* 11:67 10.1186/s13024-016-0134-z PMC5053352 27716410

[B102] Rajapaksha T. W. Eimer W. A. Bozza T. C. Vassar R. (2011). The Alzheimer’s β-secretase enzyme BACE1 is required for accurate axon guidance of olfactory sensory neurons and normal glomerulus formation in the olfactory bulb. *Mol. Neurodegener.* 6:88 10.1186/1750-1326-6-88 PMC3269394 22204380

[B103] Rochin L. Hurbain I. Serneels L. Fort C. Watt B. Leblanc P. (2013). BACE2 processes PMEL to form the melanosome amyloid matrix in pigment cells. *Proc. Natl. Acad. Sci. U.S.A.* 110 10658–10663. 10.1073/pnas.1220748110 23754390 PMC3696817

[B104] Rulifson I. C. Cao P. Miao L. Kopecky D. Huang L. White R. D. (2016). Identification of human islet amyloid polypeptide as a BACE2 substrate. *PLoS ONE* 11:e0147254 10.1371/journal.pone.0147254 PMC4739698 26840340

[B105] Rumsey J. W. Das M. Kang J. F. Wagner R. Molnar P. Hickman J. J. (2008). Tissue engineering intrafusal fibers: dose- and time-dependent differentiation of nuclear bag fibers in a defined in vitro system using neuregulin 1-beta-1. *Biomaterials* 29 994–1004. 10.1016/j.biomaterials.2007.10.042 18076984 PMC2276654

[B106] Sachse C. C. Kim Y. H. Agsten M. Huth T. Alzheimer C. Kovacs D. M. (2013). BACE1 and presenilin/gamma-secretase regulate proteolytic processing of KCNE1 and 2, auxiliary subunits of voltage-gated potassium channels. *FASEB J.* 27 2458–2467. 10.1096/fj.12-214056 23504710 PMC3659364

[B107] Sadleir K. R. Kandalepas P. C. Buggia-Prevot V. Nicholson D. A. Thinakaran G. Vassar R. (2016). Presynaptic dystrophic neurites surrounding amyloid plaques are sites of microtubule disruption, BACE1 elevation, and increased Abeta generation in Alzheimer’s disease. *Acta Neuropathol.* 132 235–256. 10.1007/s00401-016-1558-9 26993139 PMC4947125

[B108] Sannerud R. Declerck I. Peric A. Raemaekers T. Menendez G. Zhou L. (2011). ADP ribosylation factor 6 (ARF6) controls amyloid precursor protein (APP) processing by mediating the endosomal sorting of BACE1. *Proc. Natl. Acad. Sci. U.S.A.* 108 E559–E568. 10.1073/pnas.1100745108 21825135 PMC3161548

[B109] Santosa C. Rasche S. Barakat A. Bellingham S. A. Ho M. Tan J. (2011). Decreased expression of GGA3 protein in Alzheimer’s disease frontal cortex and increased co-distribution of BACE with the amyloid precursor protein. *Neurobiol. Dis.* 43 176–183. 10.1016/j.nbd.2011.03.009 21440067

[B110] Savonenko A. V. Melnikova T. Laird F. M. Stewart K. A. Price D. L. Wong P. C. (2008). Alteration of BACE1-dependent NRG1/ErbB4 signaling and schizophrenia-like phenotypes in BACE1-null mice. *Proc. Natl. Acad. Sci. U.S.A.* 105 5585–5590. 10.1073/pnas.0710373105 18385378 PMC2291091

[B111] Sharoar M. G. Yan R. (2017). Effects of altered RTN3 expression on BACE1 activity and Alzheimer’s neuritic plaques. *Rev. Neurosci.* 28 145–154. 10.1515/revneuro-2016-0054 27883331

[B112] Shi X. P. Chen E. Yin K. C. Na S. Garsky V. M. Lai M. T. (2001). The pro domain of beta-secretase does not confer strict zymogen-like properties but does assist proper folding of the protease domain. *J. Biol. Chem.* 276 10366–10373. 10.1074/jbc.M009200200 11266439

[B113] Shimizu H. Tosaki A. Kaneko K. Hisano T. Sakurai T. Nukina N. (2008). Crystal structure of an active form of BACE1, an enzyme responsible for amyloid beta protein production. *Mol. Cell. Biol.* 28 3663–3671. 10.1128/MCB.02185-07 18378702 PMC2423307

[B114] Shimshek D. R. Jacobson L. H. Kolly C. Zamurovic N. Balavenkatraman K. K. Morawiec L. (2016). Pharmacological BACE1 and BACE2 inhibition induces hair depigmentation by inhibiting PMEL17 processing in mice. *Sci. Rep.* 6:21917 10.1038/srep21917 PMC4766495 26912421

[B115] Sinha S. Anderson J. P. Barbour R. Basi G. S. Caccavello R. Davis D. (1999). Purification and cloning of amyloid precursor protein beta-secretase from human brain. *Nature* 402 537–540. 10.1038/990114 10591214

[B116] Song W. J. Son M. Y. Lee H. W. Seo H. Kim J. H. Chung S. H. (2015). Enhancement of BACE1 activity by p25/Cdk5-mediated phosphorylation in Alzheimer’s disease. *PLoS ONE* 10:e0136950 10.1371/journal.pone.0136950 PMC4552876 26317805

[B117] Stassart R. M. Fledrich R. Velanac V. Brinkmann B. G. Schwab M. H. Meijer D. (2013). A role for Schwann cell-derived neuregulin-1 in remyelination. *Nat. Neurosci.* 16 48–54. 10.1038/nn.3281 23222914

[B118] Stefansson H. Sigurdsson E. Steinthorsdottir V. Bjornsdottir S. Sigmundsson T. Ghosh S. (2002). Neuregulin 1 and susceptibility to schizophrenia. *Am. J. Hum. Genet.* 71 877–892. 10.1086/342734 12145742 PMC378543

[B119] Steuble M. Diep T. M. Schatzle P. Ludwig A. Tagaya M. Kunz B. (2012). Calsyntenin-1 shelters APP from proteolytic processing during anterograde axonal transport. *Biol. Open* 1 761–774. 10.1242/bio.20121578 23213470 PMC3507217

[B120] Stutzer I. Selevsek N. Esterhazy D. Schmidt A. Aebersold R. Stoffel M. (2013). Systematic proteomic analysis identifies beta-site amyloid precursor protein cleaving enzyme 2 and 1 (BACE2 and BACE1) substrates in pancreatic beta-cells. *J. Biol. Chem.* 288 10536–10547. 10.1074/jbc.M112.444703 23430253 PMC3624435

[B121] Tanzi R. E. Gusella J. F. Watkins P. C. Bruns G. A. St George-Hyslop P. Van Keuren M. L. (1987). Amyloid beta protein gene: cDNA, mRNA distribution, and genetic linkage near the Alzheimer locus. *Science* 235 880–884. 10.1126/science.2949367 2949367

[B122] Taveggia C. Thaker P. Petrylak A. Caporaso G. L. Toews A. Falls D. L. (2008). Type III neuregulin-1 promotes oligodendrocyte myelination. *Glia* 56 284–293. 10.1002/glia.20612 18080294

[B123] Taveggia C. Zanazzi G. Petrylak A. Yano H. Rosenbluth J. Einheber S. (2005). Neuregulin-1 type III determines the ensheathment fate of axons. *Neuron* 47 681–694. 10.1016/j.neuron.2005.08.017 16129398 PMC2387056

[B124] Tesco G. Koh Y. H. Kang E. L. Cameron A. N. Das S. Sena-Esteves M. (2007). Depletion of GGA3 stabilizes BACE and enhances beta-secretase activity. *Neuron* 54 721–737. 10.1016/j.neuron.2007.05.012 17553422 PMC1973166

[B125] Tomasselli A. G. Qahwash I. Emmons T. L. Lu Y. Leone J. W. Lull J. M. (2003). Employing a superior BACE1 cleavage sequence to probe cellular APP processing. *J. Neurochem.* 84 1006–1017. 10.1046/j.1471-4159.2003.01597.x 12603825

[B126] Treiber H. Hagemeyer N. Ehrenreich H. Simons M. (2012). BACE1 in central nervous system myelination revisited. *Mol. Psychiatry* 17 237–239. 10.1038/mp.2011.140 22042227

[B127] Turner R. T. III Loy J. A. Nguyen C. Devasamudram T. Ghosh A. K. Koelsch G. (2002). Specificity of memapsin 1 and its implications on the design of memapsin 2 (beta-secretase) inhibitor selectivity. *Biochemistry* 41 8742–8746. 10.1021/bi025926t 12093293

[B128] Udayar V. Buggia-Prevot V. Guerreiro R. L. Siegel G. Rambabu N. Soohoo A. L. (2013). A paired RNAi and RabGAP overexpression screen identifies Rab11 as a regulator of beta-amyloid production. *Cell Rep.* 5 1536–1551. 10.1016/j.celrep.2013.12.005 24373285 PMC4004174

[B129] Vagnoni A. Perkinton M. S. Gray E. H. Francis P. T. Noble W. Miller C. C. (2012). Calsyntenin-1 mediates axonal transport of the amyloid precursor protein and regulates Abeta production. *Hum. Mol. Genet.* 21 2845–2854. 10.1093/hmg/dds109 22434822 PMC3373235

[B130] van Bebber F. Hruscha A. Willem M. Schmid B. Haass C. (2013). Loss of Bace2 in zebrafish affects melanocyte migration and is distinct from Bace1 knock out phenotypes. *J. Neurochem.* 127 471–481. 10.1111/jnc.12198 23406323

[B131] Vassar R. (2014). BACE1 inhibitor drugs in clinical trials for Alzheimer’s disease. *Alzheimers Res. Ther.* 6:89 10.1186/s13195-014-0089-7 PMC4304279 25621019

[B132] Vassar R. Bennett B. D. Babu-Khan S. Kahn S. Mendiaz E. A. Denis P. (1999). Beta-secretase cleavage of Alzheimer’s amyloid precursor protein by the transmembrane aspartic protease BACE. *Science* 286 735–741. 10.1126/science.286.5440.735 10531052

[B133] Vassar R. Kovacs D. M. Yan R. Wong P. C. (2009). The β-secretase enzyme BACE in health and Alzheimer’s disease: regulation, cell biology, function, and therapeutic potential. *J. Neurosci.* 29 12787–12794. 10.1523/JNEUROSCI.3657-09.2009 19828790 PMC2879048

[B163] Vassar R. Kuhn P.-H. Haass C. Kennedy M. E. Rajendran L. Wong P. C. (2014). Function, therapeutic potential and cell biology of BACE proteases: current status and future prospects. *J. Neurochem.* 130 4–28. 10.1111/jnc.12715 24646365 PMC4086641

[B134] Vetrivel K. S. Meckler X. Chen Y. Nguyen P. D. Seidah N. G. Vassar R. (2009). Alzheimer disease Abeta production in the absence of S-palmitoylation-dependent targeting of BACE1 to lipid rafts. *J. Biol. Chem.* 284 3793–3803. 10.1074/jbc.M808920200 19074428 PMC2635050

[B135] von E. B. Wahler A. Schips T. Serrano-Pozo A. Proepper C. Boeckers T. M. (2015). The golgi-localized gamma-ear-containing ARF-binding (GGA) proteins alter amyloid-beta precursor protein (APP) processing through interaction of their GAE domain with the beta-site APP cleaving enzyme 1 (BACE1). *PLoS ONE* 10:e0129047 10.1371/journal.pone.0129047 PMC4460050 26053850

[B136] Wahle T. Prager K. Raffler N. Haass C. Famulok M. Walter J. (2005). GGA proteins regulate retrograde transport of BACE1 from endosomes to the trans-Golgi network. *Mol. Cell. Neurosci.* 29 453–461. 10.1016/j.mcn.2005.03.014 15886016

[B137] Walker K. R. Kang E. L. Whalen M. J. Shen Y. Tesco G. (2012). Depletion of GGA1 and GGA3 mediates postinjury elevation of BACE1. *J. Neurosci.* 32 10423–10437. 10.1523/JNEUROSCI.5491-11.2012 22836275 PMC3490187

[B138] Walter J. Fluhrer R. Hartung B. Willem M. Kaether C. Capell A. (2001). Phosphorylation regulates intracellular trafficking of beta-secretase. *J. Biol. Chem.* 276 14634–14641. 10.1074/jbc.M011116200 11278841

[B139] Wang C. L. Tang F. L. Peng Y. Shen C. Y. Mei L. Xiong W. C. (2012). VPS35 regulates developing mouse hippocampal neuronal morphogenesis by promoting retrograde trafficking of BACE1. *Biol. Open* 1 1248–1257. 10.1242/bio.20122451 23259059 PMC3522886

[B140] Wang H. Megill A. Wong P. C. Kirkwood A. Lee H. K. (2014). Postsynaptic target specific synaptic dysfunctions in the CA3 area of BACE1 knockout mice. *PLoS ONE* 9:e92279 10.1371/journal.pone.0092279 PMC3956924 24637500

[B141] Wang H. Song L. Laird F. Wong P. C. Lee H. K. (2008). BACE1 knock-outs display deficits in activity-dependent potentiation of synaptic transmission at mossy fiber to CA3 synapses in the hippocampus. *J. Neurosci.* 28 8677–8681. 10.1523/JNEUROSCI.2440-08.2008 18753368 PMC2728626

[B142] Wang R. Ying Z. Zhao J. Zhang Y. Wang R. Lu H. (2012). Lys(203) and Lys(382) are essential for the proteasomal degradation of BACE1. *Curr. Alzheimer Res.* 9 606–615. 10.2174/156720512800618026 22299711

[B143] Warren C. M. Kani K. Landgraf R. (2006). The N-terminal domains of neuregulin 1 confer signal attenuation. *J. Biol. Chem.* 281 27306–27316. 10.1074/jbc.M512887200 16825199

[B144] Weber M. Wu T. Meilandt W. J. Dominguez S. L. Solanoy H. O. Maloney J. A. (2017). BACE1 across species: a comparison of the in vivo consequences of BACE1 deletion in mice and rats. *Sci. Rep.* 7:44249 10.1038/srep44249 PMC5345047 28281673

[B145] Wen L. Tang F. L. Hong Y. Luo S. W. Wang C. L. He W. (2011). VPS35 haploinsufficiency increases Alzheimer’s disease neuropathology. *J. Cell Biol.* 195 765–779. 10.1083/jcb.201105109 22105352 PMC3257571

[B146] Willem M. Garratt A. N. Novak B. Citron M. Kaufmann S. Rittger A. (2006). Control of peripheral nerve myelination by the beta-secretase BACE1. *Science* 314 664–666. 10.1126/science.1132341 16990514

[B147] Wolfe M. S. De Los A. J. Miller D. D. Xia W. Selkoe D. J. (1999). Are presenilins intramembrane-cleaving proteases? Implications for the molecular mechanism of Alzheimer’s disease. *Biochemistry* 38 11223–11230. 10.1021/bi991080q 10471271

[B148] Wong H. K. Sakurai T. Oyama F. Kaneko K. Wada K. Miyazaki H. (2005). beta Subunits of voltage-gated sodium channels are novel substrates of beta-site amyloid precursor protein-cleaving enzyme (BACE1) and gamma-secretase. *J. Biol. Chem.* 280 23009–23017. 10.1074/jbc.M414648200 15824102

[B149] Xiong K. Cai H. Luo X. G. Struble R. G. Clough R. W. Yan X. X. (2007). Mitochondrial respiratory inhibition and oxidative stress elevate beta-secretase (BACE1) proteins and activity in vivo in the rat retina. *Exp. Brain Res.* 181 435–446. 10.1007/s00221-007-0943-y 17429617

[B150] Yan R. (2016). Stepping closer to treating Alzheimer’s disease patients with BACE1 inhibitor drugs. *Transl. Neurodegener.* 5:13 10.1186/s40035-016-0061-5 PMC4944430 27418961

[B151] Yan R. Bienkowski M. J. Shuck M. E. Miao H. Tory M. C. Pauley A. M. (1999). Membrane-anchored aspartyl protease with Alzheimer’s disease beta-secretase activity. *Nature* 402 533–537. 10.1038/990107 10591213

[B152] Yan R. Fan Q. Zhou J. Vassar R. (2016). Inhibiting BACE1 to reverse synaptic dysfunctions in Alzheimer’s disease. *Neurosci. Biobehav. Rev.* 65 326–340. 10.1016/j.neubiorev.2016.03.025 27044452 PMC4856578

[B153] Yan R. Han P. Miao H. Greengard P. Xu H. (2001a). The transmembrane domain of the Alzheimer’s beta-secretase (BACE1) determines its late Golgi localization and access to beta -amyloid precursor protein (APP) substrate. *J. Biol. Chem.* 276 36788–36796. 10.1074/jbc.M104350200 11466313

[B154] Yan R. Munzner J. B. Shuck M. E. Bienkowski M. J. (2001b). BACE2 functions as an alternative α-secretase in cells. *J. Biol. Chem.* 276 34019–34027. 10.1074/jbc.M105583200 11423558

[B155] Yan R. Vassar R. (2014). Targeting the beta secretase BACE1 for Alzheimer’s disease therapy. *Lancet Neurol.* 13 319–329. 10.1016/S1474-4422(13)70276-X 24556009 PMC4086426

[B156] Yeates E. F. Tesco G. (2016). The endosome-associated deubiquitinating enzyme USP8 regulates BACE1 enzyme ubiquitination and degradation. *J. Biol. Chem.* 291 15753–15766. 10.1074/jbc.M116.718023 27302062 PMC4957057

[B157] Yin D. M. Chen Y. J. Lu Y. S. Bean J. C. Sathyamurthy A. Shen C. (2013). Reversal of behavioral deficits and synaptic dysfunction in mice overexpressing neuregulin 1. *Neuron* 78 644–657. 10.1016/j.neuron.2013.03.028 23719163 PMC4176764

[B158] Yu E. J. Ko S. H. Lenkowski P. W. Pance A. Patel M. K. Jackson A. P. (2005). Distinct domains of the sodium channel beta3-subunit modulate channel-gating kinetics and subcellular location. *Biochem. J.* 392 519–526. 10.1042/BJ20050518 16080781 PMC1316291

[B159] Yu G. Nishimura M. Arawaka S. Levitan D. Zhang L. Tandon A. (2000). Nicastrin modulates presenilin-mediated notch/glp-1 signal transduction and betaAPP processing. *Nature* 407 48–54. 10.1038/35024009 10993067

[B160] Zhao Y. Wang Y. Yang J. Wang X. Zhao Y. Zhang X. (2012). Sorting nexin 12 interacts with BACE1 and regulates BACE1-mediated APP processing. *Mol. Neurodegener.* 7:30 10.1186/1750-1326-7-30 PMC3439308 22709416

[B161] Zhou L. Barao S. Laga M. Bockstael K. Borgers M. Gijsen H. (2012). The neural cell adhesion molecules L1 and CHL1 are cleaved by BACE1 protease in vivo. *J. Biol. Chem.* 287 25927–25940. 10.1074/jbc.M112.377465 22692213 PMC3406677

[B162] Zuhl A. M. Nolan C. E. Brodney M. A. Niessen S. Atchison K. Houle C. (2016). Chemoproteomic profiling reveals that cathepsin D off-target activity drives ocular toxicity of beta-secretase inhibitors. *Nat. Commun.* 7:13042 10.1038/ncomms13042 PMC5062570 27727204

